# A Novel Co-Designed Multi-Domain Entropy and Its Dynamic Synapse Classification Approach for EEG Seizure Detection

**DOI:** 10.3390/e27090919

**Published:** 2025-08-30

**Authors:** Guanyuan Feng, Jiawen Li, Yicheng Zhong, Shuang Zhang, Xin Liu, Mang I Vai, Kaihan Lin, Xianxian Zeng, Jun Yuan, Rongjun Chen

**Affiliations:** 1 School of Computer Science, Guangdong Polytechnic Normal University, Guangzhou 510665, China; fengguanyuan@gpnu.edu.cn (G.F.); zhongyicheng@stu.gpnu.edu.cn (Y.Z.); kaihanlin@gpnu.edu.cn (K.L.); zengxianxian@gpnu.edu.cn (X.Z.); yuanjun@gpnu.edu.cn (J.Y.); 2 State Key Laboratory for Novel Software Technology, Nanjing University, Nanjing 210023, China; 3 ZUMRI-LYG Joint Laboratory, Zhuhai UM Science and Technology Research Institute, Zhuhai 519031, China; fstmiv@um.edu.mo; 4 School of Artificial Intelligence, Neijiang Normal University, Neijiang 641004, China; zhang.s@njtc.edu.cn; 5 School of Life Science and Technology, University of Electronic Science and Technology of China, Chengdu 610056, China; 6 School of Mathematics and Computer Science, Northwest Minzu University, Lanzhou 730030, China; xinliu2024@xbmu.edu.cn; 7 Department of Electrical and Computer Engineering, University of Macau, Macau 999078, China; 8 Guangdong Provincial Key Laboratory of Intellectual Property and Big Data, Guangdong Polytechnic Normal University, Guangzhou 510665, China

**Keywords:** electroencephalography (EEG), dynamic synapse classifier (DySC), multi-domain entropy (MDE), seizure detection

## Abstract

Automated electroencephalography (EEG) seizure detection is meaningful in clinical medicine. However, current approaches often lack comprehensive feature extraction and are limited by generic classifier architectures, which limit their effectiveness in complex real-world scenarios. To overcome this traditional coupling between feature representation and classifier development, this study proposes DySC-MDE, an end-to-end co-designed framework for seizure detection. A novel multi-domain entropy (MDE) representation is constructed at the feature level based on amplitude-sensitive permutation entropy (ASPE), which adopts entropy-based quantifiers to characterize the nonlinear dynamics of EEG signals across diverse domains. Specifically, ASPE is extended into three distinct variants, refined composite multiscale ASPE (RCMASPE), discrete wavelet transform-based hierarchical ASPE (HASPE-DWT), and time-shift multiscale ASPE (TSMASPE), to represent various temporal and spectral dynamics of EEG signals. At the classifier level, a dynamic synapse classifier (DySC) is proposed to align with the structure of the MDE features. Particularly, DySC includes three parallel and specialized processing pathways, each tailored to a specific entropy variant. These outputs are then adaptively fused through a dynamic synaptic gating mechanism, which can enhance the model’s ability to integrate heterogeneous information sources. To fully evaluate the effectiveness of the proposed method, extensive experiments are conducted on two public datasets using cross-validation. For the binary classification task, DySC-MDE achieves an accuracy of 97.50% and 98.93% and an F1-score of 97.58% and 98.87% in the Bonn and CHB-MIT datasets, respectively. Moreover, in the three-class task, the proposed method maintains a high F1-score of 96.83%, revealing its strong discriminative performance and generalization ability across different categories. Consequently, these impressive results demonstrate that the joint optimization of nonlinear dynamic feature representations and structure-aware classifiers can further improve the analysis of complex epileptic EEG signals, which opens a novel direction for robust seizure detection.

## 1. Introduction

Neurological disorders, encompassing a broad spectrum from psychiatric conditions to neurodegenerative diseases, represent one of the most formidable challenges in global public health. These conditions not only cause long-term physical and psychological suffering for affected individuals but also impose a considerable economic and societal burden [1]. Among them, epilepsy is of particular concern due to its sudden onset and high prevalence. Currently, approximately 50 million people worldwide are affected by epilepsy, a neurological condition characterized by recurrent and unpredictable seizures, which are transient episodes of brain dysfunction resulting from abnormal and hypersynchronous discharges within cortical neuron populations [2]. Given its clinical complexity, accurate diagnosis is vital for effective treatment planning and enhancing patient outcomes and quality of life. Usually, epilepsy diagnosis relies on a combination of clinical observations and electroencephalography (EEG) monitoring. However, this traditional diagnostic approach has several limitations. Specifically, the sporadic and unpredictable nature of seizures necessitates prolonged EEG monitoring, often lasting for several hours or even days, which places a significant burden on healthcare resources. Moreover, manual EEG interpretation by neurologists is inherently time-consuming and labor-intensive. It is also susceptible to diagnostic inaccuracies due to the subjective nature of interpretation and the potential for human fatigue.

To address the challenges above, artificial intelligence (AI), particularly machine learning and deep learning, is progressively reshaping the field of medical diagnostics [3,4]. By autonomously learning complex patterns from EEG signals, AI-based systems can integrate human expertise with data-driven objectivity and computational efficiency. Hence, the integration of AI is evident in the context of epilepsy diagnosis, which beneficially analyzes EEG signals to identify abnormal neural activity, often by investigating their underlying nonlinear dynamics. It offers clinicians a robust tool for automated seizure detection and clinical decision-making support.

The effectiveness of EEG seizure detection is highly contingent upon the accurate characterization of brain activity transitions associated with seizure onset. From the nonlinear dynamics perspective, healthy or interictal EEG signals are typically complex, stochastic, and desynchronized. In contrast, during a seizure episode, large populations of neurons exhibit abnormal, highly synchronous oscillatory activity [5], resulting in a sudden transition in EEG signals from a high-dimensional, chaotic state to a low-dimensional, rhythmically pathological pattern. Morphologically, this transition is reflected in the emergence of distinctive graph elements, including high-amplitude, short-duration spikes, sharp waves, and the hallmark spike-and-wave complexes [6,7]. Hence, accurately quantifying this dynamic shift from complex disorder to pathological order represents a key challenge for achieving reliable automated seizure detection.

The typical approach to automated seizure detection generally contains two major technical paradigms: the traditional machine learning paradigm centered on feature engineering and the end-to-end deep learning methodology. In traditional feature engineering, the emphasis is placed on extracting highly discriminative features from complex EEG signals. For example, techniques such as the discrete wavelet transform (DWT) have been widely employed in the time-frequency domain to decompose signals into various frequency bands for feature extraction [8]. Adaptive approaches, such as the empirical wavelet transform (EWT), have also been applied to extract EEG brain rhythms with greater flexibility [9]. In nonlinear dynamics, entropy-based measures have demonstrated strong potential for analyzing the complexity of EEG signals. Various graph entropies derived from complex network theory have been systematically reviewed by Supriya et al. [10]. In parallel, several works have proposed a range of novel feature extraction and selection approaches, such as the distance-and-vote-based FriendPat features [11], image-based Gramian angular field (GASF) representations [12], and Hellinger distance-guided subset selection using particle swarm optimization (PSO) [13]. At the classification level, conventional machine learning classifiers, such as the support vector machine (SVM) [14], k-nearest neighbors (kNN) [15], and random forest, are commonly used due to their computational efficiency and model interpretability. However, their key limitation lies in the dependence on data-driven prior knowledge for feature extraction [16].

To overcome this drawback, end-to-end deep learning methods have emerged as a solution that automatically learns hierarchical representations directly from raw EEG recordings. Convolutional neural networks (CNNs) are popular due to their capacity to extract local patterns. A typical strategy involves transforming one-dimensional EEG signals into two-dimensional time-frequency representations, such as spectrograms [17] or continuous wavelet transform (CWT) scalograms [18], which are then processed by well-established 2D-CNN architectures like ResNet, VGGNet, and AlexNet for seizure detection. For example, Sun et al. [19] offered HG-SANet, a fusion model that utilizes the Hilbert spectrum (HS) and a self-attention mechanism to enhance seizure detection performance. Several studies applied 1D-CNN directly to retain temporal continuity, as revealed by the effectiveness through optimized 1D-CNN architectures investigated by Zaid et al. [20]. Similarly, Zhang et al. [21] applied CNN frameworks for the automated detection of interictal epileptiform discharges (IEDs), demonstrating their advances in clinical applications.

Additionally, to better analyze temporal dependencies in EEG signals, recurrent neural networks (RNNs) and their variants, such as long short-term memory (LSTM) and gated recurrent unit (GRU) networks, have been adopted. Hybrid architectures that incorporate CNNs and RNNs have also been extensively explored. For instance, CNN-LSTM and CNN-BiLSTM models proposed by Varlı and Yılmaz [22] and Cao et al. [23] integrate spatial feature extraction with sequential modeling. Meanwhile, advanced architectures, such as transformers and temporal convolutional networks (TCNs), are gaining increasing prominence. For instance, Zhu et al. [24] fused transformers with LSTM and GRU components to simultaneously represent global and local dependencies in EEG signals. Huang et al. [25] combined TCNs with self-attention mechanisms, while Peng et al. [26] developed a wavelet attention module, where these methods performed well on pediatric seizure datasets.

Furthermore, several emerging trends are shaping the development of EEG seizure detection. One is multimodal fusion, wherein EEG signals are fused with other physiological modalities, such as electrocardiography (ECG) [27] and behavioral data [28], to improve the robustness of seizure detection. Another promising direction involves graph neural networks (GNNs), which model inter-channel spatial dependencies for channel-wise causal analysis [29]. Multi-view learning also offers an advantageous framework for integrating complementary information from different feature spaces, as investigated by Fu et al. [30] and Zang et al. [31]. These approaches enhance detection performance by incorporating heterogeneous feature representations. Finally, it can be found that the success of all methods above closely depends on the quality of the input signals, making advanced EEG denoising and artifact removal techniques indispensable [32].

Although existing works have made substantial progress in automated seizure detection, a fundamental disconnect remains. Feature engineering-based methods generally offer good interpretability. However, they suffer from weak coupling between the extracted features and the classifier [33]. While end-to-end deep learning approaches are powerful, they often sacrifice interpretability, as their black-box nature raises concerns in the context of high-reliability medical diagnostics [34]. Therefore, the core objective of this study is not merely to propose a novel method but to develop an explainable AI (XAI) framework for EEG seizure detection, where the key to advancing performance lies in the tight integration between valuable feature extraction and an appropriate classifier. In summary, this study makes three main contributions to EEG seizure detection as follows:

1. A comprehensive multi-domain entropy (MDE) feature set is developed to characterize the nonlinear dynamics of EEG signals. Building upon the classical amplitude-sensitive permutation entropy (ASPE), this feature set integrates three advanced variants: refined composite multiscale ASPE (RCMASPE) for enhancing stability and noise robustness, discrete wavelet transform-based hierarchical ASPE (HASPE-DWT) to denote frequency-specific hierarchical features, and time-shift multiscale ASPE (TSMASPE) to model temporal evolution. Together, they provide a holistic representation of epileptic EEG complexity.
2. A dynamic synapse classifier (DySC) is developed according to the MDE feature to enable structure-aware classification. Particularly, it consists of three parallel and semantically distinct processing pathways tailored to individual entropy sources. Their outputs are adaptively fused through a dynamic synaptic gating mechanism, effectively integrating heterogeneous information streams.
3. Method comparisons are performed against state-of-the-art approaches using two epilepsy datasets (Bonn and CHB-MIT). The proposed DySC-MDE demonstrates satisfactory classification performance, validating the co-design idea in improving automated EEG seizure detection.

**Figure 1:**
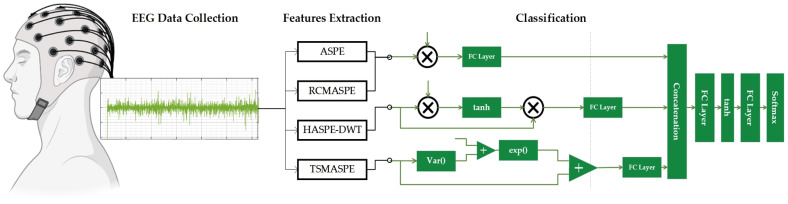
Overall workflow of the proposed DySC-MDE framework for EEG seizure detection.

For better understanding, Figure 1 illustrates the overall workflow of the proposed DySC-MDE framework, which depicts the entire processing pipeline, beginning with EEG data acquisition, followed by the core MDE feature extraction, and culminating in subsequent classification using DySC. The following sections offer a detailed description of each part.

## 2. Proposed Method

### 2.1. Datasets

The Bonn dataset [35] includes well-defined class separations and high-quality epileptic EEG recordings. Hence, it is a widely accepted benchmark for method validation and performance comparison in seizure detection. In detail, it contains five subsets (originally labeled Z, O, N, F, and S; hereafter referred to as A, B, C, D, and E for clarity), each including 100 number of single-channel EEG signals, which last 23.6 s, are sampled at 173.61 Hz, and consist of 4097 data points. Please note that this dataset exhibits a mix of modalities, specifically including both surface EEG (subsets A and B for healthy subjects) and intracranial EEG (subsets C, D, and E for epileptic subjects). While this heterogeneity presents a challenge and may be prone to selection bias in clinical contexts, this study primarily utilizes it as a benchmark to validate the proposed framework across various acquisition conditions. The description of the five subsets is listed in Table 1.

To comprehensively evaluate the seizure detection performance of the proposed framework across diverse scenarios, four tasks (denoted as cases 1–4) were formulated based on the Bonn dataset, as listed in Table 2. These tasks contain a range of detection complexities, from basic binary classifications to three-class classifications. Therefore, the DySC-MDE was applied to the four tasks, with evaluation metrics reported in Section 3.

The CHB-MIT dataset [36] is another public benchmark EEG dataset used for seizure detection, from 22 pediatric patients (5 males aged 3–22 years; 17 females aged 1.5–19 years). These records are divided into 23 cases (named chb01–chb23) and adhere to the international 10–20 system for electrode placement. Each case is recorded at a sampling rate of 256 Hz and a resolution of 16 bits. Each file has a recording duration of 1–4 h and includes 23–26 channels. The original data contains expert-annotated epileptic seizure events, with a total of 182 seizures. To ensure the validity of the EEG signals, following the previous work by Chung et al. [37], a total of 13 cases and their corresponding representative seizure locations on the scalp are assessed in this study, as shown in Table 3. Considering factors related to sample quantity and balance, healthy samples are selected in equal numbers from each case based on the number of epileptic seizure samples within that case. Meanwhile, the EEG signals are segmented into 5 min intervals, resulting in a total of 2141 samples for experimental validation.

### 2.2. MDE Feature Set

#### 2.2.1. ASPE Feature

In this study, a particular MDE feature set is developed to comprehensively analyze the complexity of epileptic EEG signals from a nonlinear dynamics perspective, which is based on ASPE [38] and integrates three complementary modified algorithms designed to investigate the signals across three key dimensions: robustness, hierarchical frequency components, and temporal structure.

ASPE is an enhanced algorithm built upon traditional permutation entropy (PE). It achieves greater sensitivity to the dynamic changes in a signal by introducing weights correlated with signal amplitude. The computation of ASPE for a time series *X* = {*x*_1_, *x*_2_, …, *x_T_*} proceeds as follows:

First, the phase space reconstruction is performed by embedding the time series into a high-dimensional space. For a given embedding dimension (*m*) and time delay (*τ*), a set of reconstructed vectors *Y_i_* is obtained as (1):

(1) $$Y_{i}=x_{i},x_{i+\tau },\ldots ,x_{i+\left(m-1\right)\tau },\;i=1,\ldots ,T-\left(m-1\right)\tau$$

Next, an amplitude weight *w_i_* is assigned to each reconstructed vector *Y_i_*, defined by the coefficient of variation of its constituent elements as (2):

(2) $$w_{i}=\frac{std\left(Y_{i}\right)}{mean\left(Y_{i}\right)}$$

This weighting ensures that vectors exhibiting larger amplitude fluctuations exert a greater influence on the subsequent entropy calculation. Following this, permutation patterns are identified by sorting the elements of each vector *Y_i_* in ascending order to determine its unique permutation pattern *π_j_*. The weighted relative frequency *p*(*π_j_*) of each permutation pattern among the *m!* possible patterns are then calculated by (3):

(3) $$p\left(\pi_{j}\right)=\frac{\sum_{i|type\left(Y_{i}\right)=\pi_{j}}w_{i}}{\sum_{i=1}^{N}w_{i}}$$

where *N* refers to the total number of reconstructed vectors.

Lastly, the ASPE is obtained as the normalized Shannon entropy computed from the weighted probability distribution of permutation patterns as (4) and (5):

(4) $$H_{ASPE}=-\sum_{j=1}^{m!}p\left(\pi_{j}\right){log}_{2}\left(p\left(\pi_{j}\right)\right)$$

(5) $$ASPE=\frac{H_{ASPE}}{{log}_{2}\left(m!\right)}$$

#### 2.2.2. RCMASPE Feature

The RCMASPE indicates signal complexity across multiple time scales while enhancing stability and noise robustness. For a given maximum scale factor *S*_max_, the RCMASPE at each scale *s* (*s* = 1, …, *S*_max_) is computed through a three-step process.

First, composite multiscale series generation is conducted. For each scale, *s* coarse-grained subsequences are derived from the original time series *X* by initiating the coarse-graining procedure at different starting points. Each data point in the *k*-th coarse-grained subsequence, denoted as *Y*^(*k*,*s*)^ (*k* = 1, …, *s*), is obtained by averaging non-overlapping segments of the original series according to (6):

(6) $$y_{j}^{\left(k,s\right)}=\frac{1}{s}\sum_{i=\left(j-1\right)s+k}^{j_{s}+k-1}x_{i}$$

This composite coarse-graining strategy comprehensively employs the original data, reducing the random errors that arise from single-window partitioning. Then, the ASPE probability distribution *P_k_* = {*p*_1_, *p*_2_, …, *p_m!_*} is obtained individually for each of the *s* coarse-grained subsequences. Subsequently, these distributions are averaged to form a refined and more stable composite probability distribution ${\bar{P}}^{\left(s\right)}$:

(7) $${\bar{P}}^{\left(s\right)}={\left\{{\bar{p}}_{j}^{\left(s\right)}\right\}}_{j=1}^{m!}=\frac{1}{s}\sum_{k=1}^{s}P_{k}$$

Finally, the entropy at scale *s* is calculated based on this averaged probability distribution by applying the normalized Shannon entropy (8):

(8) $$RCMASPE\left(s\right)=\frac{-\sum_{j=1}^{m!}{\bar{p}}_{j}^{\left(s\right)}{log}_{2}\left({\bar{p}}_{j}^{\left(s\right)}\right)}{{log}_{2}\left(m!\right)}$$

As seen, the RCMASPE is a feature vector comprising entropies computed over all scales from 1 to *S_max_*, providing a multiscale characterization of signal complexity.

#### 2.2.3. HASPE-DWT Feature

The HASPE-DWT is applied to analyze the hierarchical complexity of EEG signals from the perspective of different frequency bands. In this approach, the original signal *X* is decomposed through an *L*-level DWT using a specific wavelet basis db4, producing one set of approximation coefficients *A_L_*, representing the lowest frequency components, and *L* sets of detail coefficients (*D_L_*, *D_L_*_−1_, …, *D*_1_), which correspond to progressively higher frequency bands. Each coefficient set (*A_L_* and each *D_i_*) is then subjected to an inverse wavelet transform to reconstruct the signal components within their respective frequency bands, denoted as *S*(*A_L_*) and *S*(*D_i_*). Subsequently, the ASPE is calculated for each reconstructed band. Hence, the HASPE-DWT yields an *L* + 1-dimensional feature vector that characterizes the hierarchical complexity structure of the EEG signals across the full spectrum (9):

(9) $$HASPE-DWT=\left[ASPE\left(S\left(A_{L}\right)\right),ASPE\left(S\left(D_{L}\right)\right),\ldots ,ASPE\left(S\left(D_{1}\right)\right)\right]$$

#### 2.2.4. TSMASPE Feature

To investigate deterministic patterns that occur at various time delays within the EEG signals, the TSMASPE is applied in this study. For a specified maximum scale factor *K_max_*, TSMASPE is obtained at each scale *k* (*k* = 1, …, *K_max_*) as follows:

First, for the current scale *k*, time-shifted subsequences are generated by sampling the original signals at intervals of length *k*. Then, the *j*-th time-shifted subsequence $Y^{\left(j,k\right)}$ (*j* = 1, …, *k*) is obtained as (10):

(10) $$Y^{\left(j,k\right)}={\{x}_{j},x_{j+k},x_{j+2k},\ldots \}$$

Next, the ASPE is calculated for each of these subsequences. The final entropy value at scale k is acquired by averaging the ASPE values across all k subsequences as (11):

(11) $$TSMASPE\left(k\right)=\frac{1}{k}\sum_{j=1}^{k}ASPE\left(Y^{\left(j,k\right)}\right)$$

The TSMASPE is a feature vector in which each element reflects the average signal complexity over different time-lag scales, which facilitates the detection of potential periodicities and long-range correlations within the EEG signals.

### 2.3. DySC Classifier

Following the construction of a dimensionally heterogeneous MDE feature set, the critical challenge lies in designing a classifier that effectively interprets and utilizes the intrinsic logic embedded within these features. Conventional machine learning models treat inputs as homogeneous vectors, such as SVM and standard multilayer perceptrons (MLPs). Although broadly applicable, they inevitably discard valuable grouping information and structural relationships among features, particularly neglecting the distinctions between features derived from scale analysis and those from hierarchical analysis. To this end, a novel classification architecture is co-designed with the MDE feature set, named DySC. This novel co-design idea is fundamentally rooted in ensuring that the classifier architecture is explicitly aligned with the underlying logic and physical meaning of its input features. It involves two primary aspects. One is semantic specialization at MDE feature extraction, where those features are meticulously designed to extract distinct nonlinear dynamic characteristics across multiple domains, including scale, hierarchy, and temporal stability, with each variant quantifying a specific aspect of EEG complexity. Another is structural alignment in the classifier, where DySC is designed with parallel and semantically distinct processing pathways, each specifically tailored to the unique nature of its corresponding entropy variant. Rather than seeking complex shared learning across raw feature spaces, the architecture choice to isolate these features into specialized branches for initial processing, which aims to preserve semantic information by avoiding information entanglement, enhance interpretability by allowing clearer attribution of decisions to specific feature types, and enable targeted modulation through dynamic gating mechanisms within such specialized pathways. Therefore, this hierarchical fusion process, from pathway-specific compression to final cross-pathway integration, is designed to utilize complementary information while respecting the inherent structural differences among feature domains.

Furthermore, this design is also biomimetic-based, embodied in two principal aspects. One is functional specialization, which involves drawing an analogy to the distinct functional areas of the cerebral cortex responsible for processing sensory modalities, such as vision or hearing, since DySC incorporates functionally specialized pathways tailored to different categories of entropy features. Another is dynamic plasticity, which is inspired by the variable synaptic strengths modulated by neural activity in biological systems. Thus, DySC employs dynamic synapses whose effective connection weights are adaptable and can be modulated instantaneously in response to the specific characteristics of the input signals.

**Figure 2:**
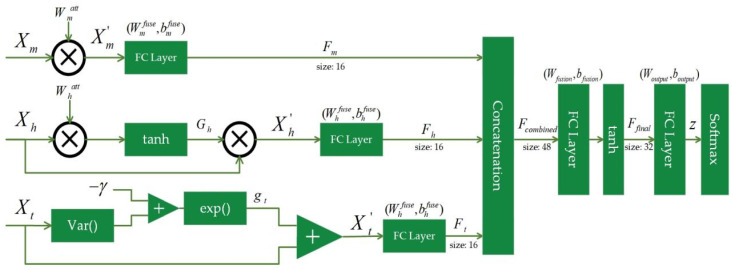
The details of the proposed DySC classifier.

In detail, the DySC classifier mainly includes three parallel processing pathways for handling multi-scale, hierarchical, and time-invariant features. The core of each pathway incorporates an individual dynamic gating mechanism. Feature representations are fused within their respective pathways, then integrated through a cross-pathway fusion layer, and ultimately passed to a softmax layer to generate the final classification output. As illustrated in Figure 2, the information processing flow of DySC is organized into four stages: structured input and parallel pathways, dynamic synapses, hierarchical information fusion, and the output layer. This architecture, while employing relatively shallow fully connected layers, is specifically co-designed to adopt the MDE feature set, focusing on targeted processing and interpretability rather than end-to-end feature learning from epileptic EEG signals.

To preserve and fully exploit the intrinsic structure of the MDE feature set, features are categorized according to their origins and physical meanings and then routed into three functionally specialized processing pathways. Here, each pathway independently handles a distinct category of features, enabling targeted and efficient processing that is aligned with its characteristics. Specifically, processing features are extracted from three domains, denoted as $X_{m}\in R^{d_{m}}$, $X_{h}\in R^{d_{h}}$, and $X_{t}\in R^{d_{t}}$, which consist of {ASPE, RCMASPE}, HASPE-DWT, and TSMASPE, respectively. This stage marks the most fundamental difference between DySC and conventional neural networks, where in each pathway, the information flow is nonlinearly modulated by an input-dependent dynamic gating mechanism.

In the hierarchical pathway, using frequency-sensitive gating to simulate the brain’s attention to specific frequency-band events (like high-frequency spikes during a seizure), the gating strength of this pathway is dynamically determined by the values of the HASPE-DWT feature vector *X_h_* itself. A gating vector *G_h_* is computed by (12):

(12) $$G_{h}=tanh\left(X_{h}⊙W_{h}^{att}\right)$$

where $W_{h}^{att}$ refers to a learnable attention weight vector with the same dimensions as *X_h_*, it is optimized during the training phase through end-to-end learning. ⊙ represents element-wise multiplication (Hadamard product). The modulated output of the pathway $X_{h}^{\prime }$ is obtained by multiplying the original input by the gating signals (13):

(13) $$X_{h}^{\prime }=X_{h}⊙G_{h}$$

This mechanism allows EEG signals from frequency bands exhibiting task-relevant changes in entropy to be dynamically amplified or highlighted, representing an adaptive process that responds to the specific characteristics of each input signal. Specifically, the $W_{h}^{att}$ reflects the saliency assigned by the model to different frequency components of the HASPE-DWT feature, which contributes to the interpretability of this pathway by indicating which frequency bands are most discriminative for seizure detection.

The time-invariant pathway adopts stability-sensitive gating to find stable patterns in the EEG signals, where the gate for this pathway is a scalar gain (*g_t_*), controlled by the overall variance of the TSMASPE feature vector *X_t_* as (14):

(14) $$g_{t}=exp\left(-\gamma \cdot Var\left(X_{t}\right)\right)$$

where *γ* means a learnable positive scalar sensitivity parameter, which can be optimized during the training phase. When the variance of the input vector *X_t_* is small (indicating a highly consistent and stable pattern across different time lags), *g_t_* approaches 1, allowing the signals to pass through completely. Conversely, when the *X_t_* is large, *g_t_* approaches 0, suppressing the signals. The pathway’s output $X_{t}^{\prime }$ is acquired by (15):

(15) $$X_{t}^{\prime }=X_{t}\cdot g_{t}$$

The stability-sensitive gating intrinsically indicates the model’s reliance on patterns that exhibit temporal consistency, which is beneficial for distinguishing pathological rhythmic activity during seizures, offering a degree of inherent interpretability regarding the model’s focus on temporal stability. Therefore, the dynamic aspect of such gating mechanisms is that their effective connection weights are adaptable in response to the specific characteristics of the input EEG signals, based on parameters learned during the training phase. This biomimetic design is inspired by how synaptic strengths in biological systems are modulated (learned during development/training) and then applied dynamically based on incoming neural activity.

The multi-scale pathway employs a relatively simple learnable static attention mechanism. It learns an input-independent weight mask $W_{m}^{att}$ that, after training, offers scales consistently proven to be essential for seizure detection. This design inherently reflects the saliency of specific temporal scales of complexity derived from RCMASPE, contributing to the model’s interpretability by identifying consistently vital scales. For an input $X_{m}$, the output $X_{m}^{\prime }$ is computed by (16):

(16) $$X_{m}^{\prime }=X_{m}⊙W_{m}^{att}$$

After modulation by the dynamic gates, the information enters a two-stage fusion process. First, the modulated output of each pathway ($X_{m}^{\prime }$, $X_{h}^{\prime }$, and $X_{t}^{\prime }$) is fed into its independent, fully connected network to extract and compress the core information within that domain. Concerning its implementation, the hyperbolic tangent (tanh) function is used as the activation function for these layers (17):

(17) $$F_{h}=tanh\left(X_{h}^{\prime }W_{h}^{fuse}+b_{h}^{fuse}\right)$$

where $W_{h}^{fuse}$ and $b_{h}^{fuse}$ are the learnable parameters of this pathway’s fusion layer.

Subsequently, the compact feature representations from the three pathways (*F_m_*, *F_n_*, and *F_t_*) are concatenated to generate a global feature vector *F_combined_* = [*F_m_*, *F_n_*, *F_t_*], which is then fed into a deep fully connected network for final cross-domain information integration.

The fusion layer connects to a standard softmax output layer, which converts the network’s output logits (*z*) into posterior probabilities (*P*) for the *K* classes as (18):

(18) $$P\left(y=j|z\right)=\frac{e^{z_{j}}}{\sum_{k=1}^{K}e^{z_{k}}}$$

Lastly, all learnable parameters of the proposed model, including the attention and gating parameters for each pathway, as well as the weights and biases of the fusion layers, are optimized end-to-end by minimizing the cross-entropy loss function (19):

(19) $$L=-\frac{1}{N_{samples}}\sum_{i=1}^{N_{samples}}\sum_{j=1}^{K}y_{i,j}log\left(p_{i,j}\right)$$

where *N*_samples_ means the total number of samples, *y_i_*_,_*_j_* denotes the one-hot encoded true label for sample *i*, and *p_i_*_,_*_j_* is the predicted probability of sample *i* belonging to class *j*. Additionally, the quasi-Newton method is employed as the optimizer to solve the minimization problem.

### 2.4. Evaluation Metrics

Based on the confusion matrix generated in each case, five widely used evaluation metrics are acquired by (20)–(24), where *TP*, *TN*, *FP*, and *FN* denote true positives, true negatives, false positives, and false negatives, respectively:

(20) $$Accuracy=\frac{TN+TP}{TN+FN+TP+FP}$$

(21) $$Precision=\frac{TP}{TP+FP}$$

(22) $$Recall=\frac{TP}{TP+FN}$$

(23) $$Specificity=\frac{TN}{TN+FP}$$

(24) $$\mathrm{F1-score}=2\times \frac{Precision\times Recall}{Precision+Recall}$$

In this study, to ensure an unbiased method evaluation, 10-fold and 20-fold cross-validation are adopted. Taking the 10-fold as an example, the entire dataset for each detection task is randomly and evenly partitioned into 10 subsets. In each iteration, one subset serves as the test set, while the remaining nine are used for training. This process is repeated ten times, allowing each subset to act as the test set exactly once. For instance, considering case 1 (A vs. E) in the Boon dataset, it comprises 200 samples (100 positive and 100 negative). In the first fold, the initial 10 positive samples and the initial 10 negative samples are allocated as the test set, with the remaining 180 samples used for training. For the second fold, the subsequent 10 positive samples (i.e., samples 11–20) and the subsequent 10 negative samples (i.e., samples 11–20) are employed as the test set, and so forth, until each sample has served as part of the test set exactly once across the ten iterations. Both 10-fold and 20-fold exhibit their balance of computational efficiency and performance estimation compared to more computationally intensive methods, such as leave-one-out cross-validation, especially given the dataset’s size and its role as a benchmark for comparison with related works.

### 2.5. Experimental Configurations

All experiments were conducted using MATLAB R2022b. The core parameter configurations for extracting the MDE feature set and the hyperparameter settings for the DySC classifier are presented in Table 4 and Table 5, respectively.

## 3. Experimental Results

### 3.1. Bonn Dataset Results

The assessment of the experiential results begins with overall performance metrics obtained through 10-fold and 20-fold cross-validations. The detailed analysis and interpretation of the results for each case are then provided, followed by an insightful investigation supported by confusion matrices that comprehensively evaluate the effectiveness and robustness of the proposed method. First, Table 6 and Table 7 present the evaluation metrics for each seizure detection task.

The results shown in Table 6 and Table 7 show that the proposed DySC-MDE framework exhibits impressive performance across all four cases for different cross-validation folds. Meanwhile, the relatively small standard deviations also indicate a stable performance. Considering Table 6, as for case 1, the model achieves an accuracy of 96.50% and an F1-score of 97.01%. The F1-score, representing the harmonic mean of precision and recall, indicates that the model balances sensitivity to seizure events with a low false positive rate for healthy samples, which demonstrates that the MDE feature set, in conjunction with the DySC, beneficially distinguishes the underlying differences between normal and epileptic EEG signals.

Then, regarding case 2, i.e., distinguishing between interictal and ictal states within the same patient, the model reveals improved performance, achieving an accuracy of 97.50% and an F1-score of 97.58%. The relatively small standard deviations observed further underscore the high reliability of the method, particularly when classifying states originating from a common pathological condition but representing different stages of progression.

Next, case 3 is a class-imbalanced binary task (400 non-ictal vs. 100 ictal samples) that poses greater challenges to model robustness. The model accomplishes an accuracy of 96.80% and a specificity of 95.03%. High specificity indicates a low false positive rate, meaning that the model accurately identifies most non-ictal samples. This result is vital in clinical contexts to mitigate patient and clinician anxiety due to frequent false alarms. The low standard deviations here also indicate that the model maintains consistent performance even under class imbalance.

Case 4 is a three-class task that requires fine-grained discrimination among three neurological states. The model achieves an accuracy of 93.80% and an F1-score of 96.83%. Interestingly, this F1-score surpasses that of case 3. A possible explanation is that subdividing the non-ictal group into healthy and interictal classes helps the model establish clearer decision boundaries, which can prevent the conflation of distinct functional states. While case 4 exhibits slightly larger standard deviations for accuracy (±5.85%) compared to other binary tasks, the F1-score’s deviation (±2.90%) remains relatively low, indicating a robust balance between precision and recall even for the complex multi-class problem.

**Figure 3:**
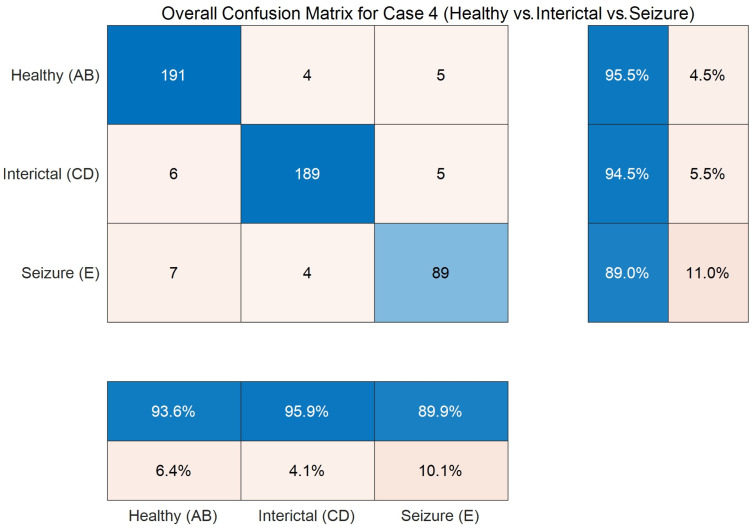
Confusion matrix on the three-class task (Boon dataset) through 10-fold cross-validation.

As seen, Table 7 exhibits a similar trend to Table 6, so now, to gain a more detailed understanding of the model’s classification dynamics, an analysis is conducted using intermediate results collected during the 10-fold cross-validation process. Particularly, for case 4, the cumulative confusion matrix across all folds is aggregated and visualized, as shown in Figure 3, which highlights the distribution of correct and incorrect predictions across the three classes, allowing for a deeper evaluation of the model’s discriminative capability.

In Figure 3, the values along the main diagonal of the confusion matrix (191, 189, and 89) are substantially higher than the off-diagonal entries, indicating strong overall detection performance across all three classes. Regarding row-wise normalized recall, the detection accuracies for the healthy (AB) and interictal (CD) classes achieve 95.5% and 94.5%, respectively. More importantly, analysis of the off-diagonal elements reveals insights into the method’s weaknesses. The primary confusion is observed between the healthy (AB) and interictal (CD) classes. For instance, six samples from the healthy class are misclassified as interictal. This result is clinically plausible, as both categories represent non-ictal states and exhibit signal characteristics more similar to those of the ictal (E) class. The recall of the clinically significant ictal (E) class is 89.0%, indicating that 11.0% of seizure samples are missed. However, regarding column-wise precision, the ictal class achieves a precision of 89.9%. It reveals that nearly nine out of ten samples predicted as seizures are true positives, demonstrating strong predictive reliability accordingly.

**Figure 4:**
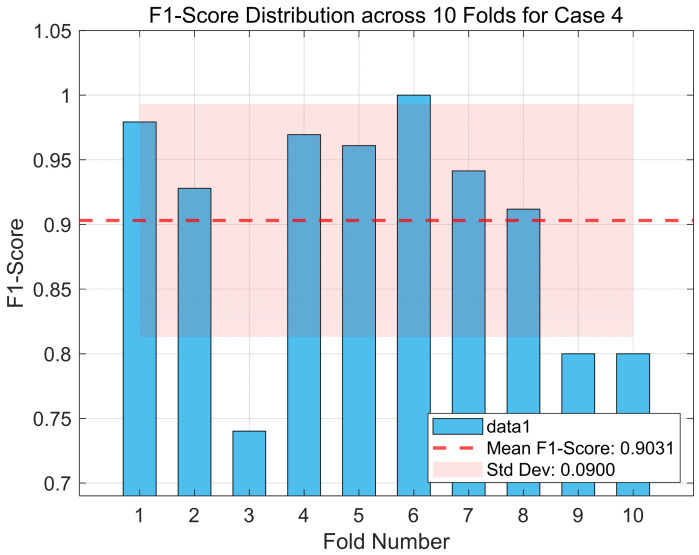
The F1-score distribution across 10 folds for case 4 (Boon dataset).

Additionally, Figure 4 displays the distribution of F1-scores obtained from the 10-fold cross-validation for case 4 of the Bonn dataset, assessing the consistency and robustness of the proposed DySC-MDE framework under varying sample partitions.

In Figure 4, although the average F1-score across the 10 folds achieves a high level (as indicated by the dashed line), the results from individual folds exhibit variations (standard deviation = 0.09). For instance, the model’s performance approaches perfection in the sixth fold (F1-score ≈ 1.0), decreasing markedly in the third, ninth, and tenth folds (F1-score ≈ 0.74–0.80). This observation suggests that, although the DySC-MDE framework performs well overall, it is sensitive to the specific partitioning of the training data. Identifying challenging samples can contribute to performance degradation, and refining feature representations or classifier design to improve robustness in these cases will be an important direction for future work. Despite these observed fluctuations across individual folds, it is noteworthy that the model’s lower performance bound remains within acceptable limits for a clinical application, and its average performance is highly competitive. The relatively low standard deviation (0.0900) across the 10 folds, coupled with the high average F1-score, reinforces the overall stability and generalization capability of the proposed method.

In short, the Bonn dataset results indicate that the proposed framework achieves high accuracy, F1-scores, and specificity across a range of binary and three-class seizure detection tasks while demonstrating strong performance stability. Further analysis of the confusion matrices reveals the model’s ability to distinguish between critical classes reliably. These impressive findings substantiate the underlying co-design idea, showing that robust automated seizure detection can be achieved by integrating a comprehensive MDE feature set with a structurally aligned DySC classifier.

### 3.2. CHB-MIT Dataset Result

To further validate the DySC-MDE framework on a more complex dataset, an additional experiment is conducted on the CHB-MIT dataset. Compared to the Bonn dataset, the CHB-MIT dataset contains long-duration EEG recordings from different pediatric patients. Similarly, the performance is also evaluated using 10-fold and 20-fold cross-validation, as displayed in Table 8.

In Table 8, the performance on the CHB-MIT dataset is also satisfactory. Regarding the 10-fold cross-validation, the DySC-MDE framework achieves an accuracy of 98.88% and an F1-score of 98.75%. These results indicate that the proposed method maintains a high level of performance even when facing a complex dataset that includes various age groups of patients and longer durations. Compared to the 10-fold, the results from the 20-fold show a slightly higher accuracy and F1-score of up to 98.93% and 98.87%, respectively, which further validates the robustness of the model under different data partitioning schemes.

**Figure 5:**
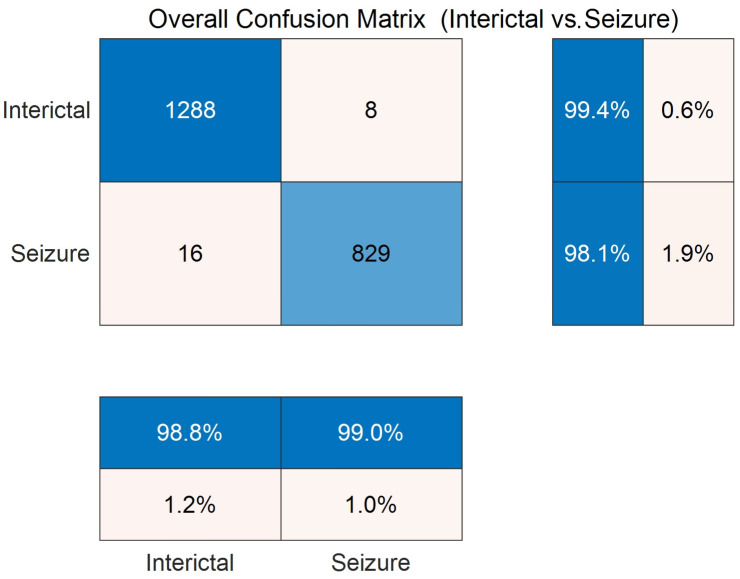
Confusion matrix on the binary classification task (CHB-MIT dataset) through 10-fold cross-validation.

Figure 5 shows the confusion matrix of the DySC-MDE framework on the CHB-MIT dataset through the 10-fold cross-validation. The diagonal elements that represent the number of correctly classified samples are significantly higher than the off-diagonal elements (1288 vs. 8, 829 vs. 16), which further validates the model’s strong classification capability. Particularly, the model performed remarkably in terms of recall and precision for the seizure class, with values of 98.1% and 99.0%, respectively, indicating it can detect true seizure events while maintaining a low false positive rate, which is beneficial for clinical applications.

**Figure 6:**
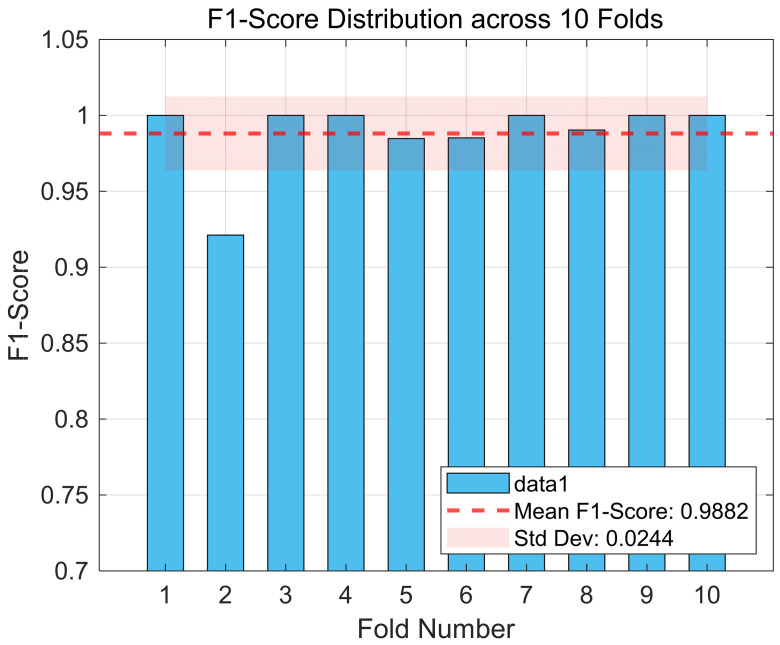
The F1-score distribution across 10 folds (CHB-MIT dataset).

Moreover, Figure 6 illustrates the distribution of the F1-scores across different folds in the 10-fold cross-validation, revealing the framework’s performance consistency under various sample partitions. As seen, the F1-scores for all folds are above 90%, and the average value (indicated by the dashed line) is very close to 100%. Although there exists a performance fluctuation, the standard deviation (±0.0244) is relatively small. This finding reveals that despite the greater heterogeneity of the CHB-MIT dataset, the overall performance of the DySC-MDE framework remains stable and does not show a significant performance degradation due to specific data partitioning, as observed in case 4 of the Bonn dataset (Figure 4).

In short, the overall results on the CHB-MIT dataset are consistent with the findings from the Bonn dataset. That implies adopting the co-design approach, i.e., integrating the MDE feature set with a structure-aware DySC classifier, can appropriately process EEG signals from different sources and with various features for achieving high performance toward automated seizure detection.

## 4. Discussion

To further contextualize the DySC-MDE framework and assess its advantages in seizure detection, particularly in terms of its approach to nonlinear dynamics, an in-depth comparative analysis is conducted with several state-of-the-art methods. Please note that all comparative results presented in Table 9, Table 10, Table 11 and Table 12 are obtained by evaluating the respective methods on the Bonn dataset through 10-fold cross-validation, while Table 13 lists the comparative study with recent works on the CHB-MIT dataset through 20-fold cross-validation, which ensures a fair comparison under the same data conditions.

First, from Table 9, Table 10, Table 11 and Table 12, DySC-MDE demonstrates highly competitive performance across all four detection tasks in the Boon dataset. Particularly in the binary scenarios of A vs. E (case 1) and C vs. E (case 2), it achieves top-tier results in key metrics, with an average accuracy exceeding 97%. Even under the three-class task, AB vs. CD vs. E (case 4), the model maintains an accuracy of 93.8% alongside a high F1-score of 96.83%. The Bonn dataset is a widely accepted benchmark in the field of EEG seizure detection, as it features well-defined class separations and high-quality epileptic EEG recordings. While it is limited in size and heterogeneity (mixing surface and intracranial EEG), and its 10-fold and 20-fold cross-validation, ordinary in existing works for this dataset, do not explicitly enforce patient-wise separation, potentially leading to correlated snippets between training and test folds from the same limited set of subjects, which is a recognized limitation in assessing generalization to new patients, its consistent use in numerous prior studies makes it a valuable standard for validating novel methodologies. The proposed framework’s highly competitive performance is attributed not to any singular algorithmic innovation but rather to the core co-design idea. This approach is particularly well-suited for analyzing EEG signals, which are known to exhibit complex nonlinear dynamics.

Second, the completeness of the feature system serves as the cornerstone of accurate detection. Unlike most studies that rely on a single feature type, the MDE feature set integrates the foundational ASPE with three complementary extensions: RCMASPE, HASPE-DWT, and TSMASPE. This comprehensive set of entropy-based features offers a holistic representation of epileptic EEG complexity, capturing scale variation, hierarchical rhythmic structure, and temporal stability. Such information forms a robust input for various seizure detection tasks. In contrast to generic black-box deep learning models, the proposed DySC classifier conducts a parallel pathway architecture that facilitates specialized processing of entropy-based features from distinct domains. For instance, the hierarchical pathway decodes frequency band information derived from HASPE-DWT, while the time-invariant pathway focuses on capturing temporal pattern stability identified by TSMASPE. This tailored architecture avoids information entanglement and feature dilution, which are common when heterogeneous inputs are flattened and processed indiscriminately. Meanwhile, the dynamic mechanism constitutes a central innovation of the proposed framework. Thus, DySC’s advantage lies in its dynamic synapse mechanism, which adaptively adjusts processing weights in real-time based on input characteristics, such as entropy fluctuations in specific frequency bands and global pattern variance. Compared with the fixed-weight structure of conventional neural networks, this input-dependent processing helps the model to operate with greater adaptability to the inherently dynamic and non-stationary EEG signals.

Third, regarding the novelty of the proposed method, compared to time-frequency image-based deep learning methods, they have employed a strategy of converting 1D signals into 2D time-frequency representations, which are then processed using CNN architectures. These methods have achieved impressive results by using the strong image feature extraction capabilities of pre-trained CNNs. However, the signal-to-image transformation may obscure fine-grained, transient, and nonlinear dynamic information inherent in the time series data, like EEG signals. In contrast, the MDE feature set quantifies complexity, hierarchical structure, and temporal dynamics from the 1D signals, which preserves the intrinsic nonlinear dynamic characteristics of the epileptic EEG signals. The DySC-MDE framework’s ability to achieve satisfactory results, such as 96.5% accuracy on case 1 (A vs. E), without requiring image conversion, suggests that deeply structured modeling of nonlinear dynamic features also offers a practical approach to EEG seizure detection.

Next, compared to feature-fusion-based hybrid deep learning methods, which integrate multiple feature types utilizing hybrid networks such as CNN-BiLSTM, they accomplish high performance through sequential feature extraction, selection, and classification stages. Nonetheless, such stages remain loosely coupled. Specifically, while these models usually employ deeper or recurrent architectures to capture temporal dependencies directly from EEG signals, the DySC classifier adopts relatively shallow fully connected layers without explicit recurrent or transformer-based temporal modeling. For instance, the hierarchical pathway is specialized for processing frequency band information from HASPE-DWT, whereas the time-invariant pathway targets temporal stability features from TSMASPE. This structured framework enables the classifier to efficiently interpret and adopt the pre-extracted nonlinear dynamic features, rather than learning them from scratch. Consequently, the DySC-MDE framework addresses the critical need for explainability in medical diagnostics. Unlike black-box deep learning models that achieve high performance at the cost of opacity, the co-design idea is designed to provide interpretability through semantically MDE features and structure-aware processing pathways with dynamic gating mechanisms. This intrinsic interpretability aligns with recent advancements in XAI, as claimed by Mazurek et al. [41]. By integrating performance with built-in interpretability, DySC-MDE offers a promising paradigm for decoding complex EEG signals where understanding the underlying patterns is often as crucial as diagnostic accuracy. It is also found that integrating DySC with deeper architectures or explicit temporal modeling techniques after feature extraction could be an interesting avenue for future investigation, which potentially further enhances performance for EEG-based diagnostics.

Moreover, Table 13 presents a comparative study with recent works on CHB-MIT. Chuang et al. [37] applied a stacked 2D-CNN with different channel settings. Their 18-channel model achieved 98.47% accuracy but lacked good results in the F1-score. Meanwhile, their four-channel and single-channel methods showed performance degradation, highlighting the trade-off between channel complexity and detection robustness. While the proposed DySC-MDE outperforms most of the recent works, achieving 98.93% accuracy, 98.94% precision, 98.87% recall, 98.89% specificity, and 98.87% F1-score. The remarkable performance stems from two key aspects. On the one hand, the MDE feature set captures the fine-grained nonlinear dynamics of EEG signals across temporal, spectral, and spatial domains, which is more adaptive to the heterogeneous patterns in CHB-MIT, such as inter-patient variability and artifact interference. On the other hand, the DySC is inherently designed to align with the characteristics of MDE, enabling efficient information fusion and discriminative learning without relying on manual feature engineering. Notably, methods like RNN-based models by Shah et al. [39] and SE-TCN-BiGRU (with/without noise) by Zhu et al. [40] offered lower accuracy and F1-score, indicating their vulnerability to noise and complex temporal dependencies in long-term EEG recordings. In this regard, DySC-MDE maintains robust performance, revealing that it bridges the gap between feature expressiveness and classifier adaptability, outperforming mainstream deep-learning pipelines. This consistency across datasets (Bonn and CHB-MIT) proves that the co-designed idea is not only effective for controlled experimental settings but also scalable to real-world clinical data, laying a solid foundation for designing epilepsy monitoring systems.

Finally, in future work, by analyzing the weights of the various pathways and the activation strengths of the dynamic gates in the trained model, which class of physiological information the model relies on most when identifying different epileptic states could be investigated, i.e., how changes in multiscale complexity, hierarchical rhythms, or temporal stability, as characterized by nonlinear dynamic measures like entropy, contribute to the seizure detection. This issue holds potential for understanding the pathophysiology of epilepsy from a nonlinear dynamics perspective. Additionally, the standard deviations in the cross-validation indicate that the proposed framework is not sensitive to data partitioning and possesses good generalization ability and stability, a vital prerequisite for developing reliable clinical decision support tools. Hence, an investigation into its hardware deployment on portable medical devices will be performed.

## 5. Conclusions

To effectively utilize the heterogeneous and nonlinear dynamic characteristics inherent in epileptic EEG signals, a novel DySC-MDE framework based on the co-design idea has been proposed in this study. It facilitates a comprehensive characterization of EEG signals across four vital dimensions: scale, robustness, hierarchy, and temporality. Therefore, such entropy-based features beneficially analyze the complex behaviors of EEG signals. Method evaluation on the Bonn and CHB-MIT datasets demonstrates that DySC-MDE accomplishes impressive results on key metrics such as accuracy and F1-score, confirming the framework’s ability to find heterogeneous patterns like inter-patient variability and artifact interference, further validating its robustness and potential for clinical translation. In the future, several advanced modules with data-driven properties [42,43,44,45] will be considered to improve the DySC-MDE and then validate it in more recent datasets such as HED [46] and SzCORE [47]. Overall, it can be said that the most significant contribution of this study lies in its robust empirical validation of the principle of co-designing feature systems and classifier architectures, where its balanced integration of performance and interpretability, driven by the entropy-based analysis of nonlinear dynamics, offers a promising paradigm for those works in complex EEG signals processing.

## Figures and Tables

**Table 1:** Key information of the Bonn epilepsy dataset.

Subset	Subject	Data Size	Description
A	Healthy	100	Surface EEG, eyes open
B	Healthy	100	Surface EEG, eyes closed
C	Epileptic	100	Intracranial EEG, hippocampal
D	Epileptic	100	Intracranial EEG, epileptic focus
E	Epileptic	100	Intracranial EEG, hippocampal focus (seizure)

**Table 2:** Four tasks (cases 1–4) in EEG seizure detection for method evaluation.

Case	Task	Clinical Significance
Case 1	A vs. E	Distinguishing between healthy individuals and the state of epileptic seizures is the basis for epilepsy diagnosis.
Case 2	C vs. E	Distinguishing between the ictal (seizure) and interictal (between seizures) periods in the same patient is vital for epilepsy early warning and real-time monitoring.
Case 3	ABCD vs. E	Distinguishing all non-ictal states (including healthy and interictal states) from the ictal state is a key task in designing a reliable seizure detection system.
Case 4	AB vs. CD vs. E	Distinguishing the three core physiological states finely is required for the proposed method to demonstrate its satisfactory discriminative ability.

**Table 3:** The selected cases and corresponding channels from the CHB-MIT dataset.

**Case**	chb01	chb02	chb03	chb04	chb05	chb07	chb08	chb10	chb11	chb15	chb17	chb22	chb23
**Channel**	P8-O2	P7-O1	Fp1-F3	P8-O2	P7-O1	Fp1-F3	Fp1-F3	P7-O1	P7-O1	P7-O1	P8-O2	Fp1-F3	Fp1-F3

**Table 4:** Core parameter configurations for the MDE feature extraction.

Feature	Parameter	Value
ASPE	Embedding dimension (*m*)	3
	Time delay (*τ*)	1
RCMASPE	Maximum scale factor (*S*_max_)	10
HASPE-DWT	Wavelet basis function	db4
	Decomposition level (*L*)	4 (Bonn) 5 (CHB-MIT)
TSMASPE	Maximum time-lag factor (*K*_max_)	10

**Table 5:** Hyperparameter settings for the DySC classifier.

Hyperparameter	Value
hidden_size_pathway	16
hidden_size_fusion	32
Cross-entropy loss function	Quasi-Newton method
MaxIterations	50
MaxFunction evaluations	1,000,000

**Table 6:** EEG seizure detection performances (mean ± standard deviation %) using the proposed method through 10-fold cross-validation (Boon dataset).

Metrics	A vs. E (Case 1)	C vs. E (Case 2)	ABCD vs. E (Case 3)	AB vs. CD vs. E (Case 4)
Accuracy (%)	96.50 ± 4.12	97.50 ± 2.64	96.80 ± 2.53	93.80 ± 5.85
Precision (%)	96.36 ± 4.35	97.30 ± 3.09	96.24 ± 4.76	92.47 ± 8.63
Recall (%)	97.01 ± 3.41	97.58 ± 2.72	94.47 ± 4.37	92.69 ± 8.64
Specificity (%)	96.41 ± 4.22	97.29 ± 2.88	95.03 ± 3.98	92.38 ± 8.56
F1-score (%)	97.01 ± 3.41	97.58 ± 2.72	94.47 ± 4.37	96.83 ± 2.90

**Table 8:** EEG seizure detection performances (mean ± standard deviation %) using the proposed method through 10-fold and 20-fold cross-validation (CHB-MIT dataset).

Metrics	10-Fold Cross-Validation	20-Fold Cross-Validation
Accuracy (%)	98.88 ± 2.30	98.93 ± 2.47
Precision (%)	98.92 ± 2.15	98.94 ± 2.47
Recall (%)	98.75 ± 2.64	98.87 ± 2.54
Specificity (%)	98.82 ± 2.44	98.89 ± 2.51
F1-score (%)	98.75 ± 2.64	98.87 ± 2.54

**Table 7:** EEG seizure detection performances (mean ± standard deviation %) using the proposed method through 20-fold cross-validation (Boon dataset).

Metrics	A vs. E (Case 1)	C vs. E (Case 2)	ABCD vs. E (Case 3)	AB vs. CD vs. E (Case 4)
Accuracy (%)	95.50 ± 5.10	97.50 ± 5.50	96.60 ± 3.25	91.60 ± 5.79
Precision (%)	94.47 ± 7.43	97.83 ± 4.72	95.07 ± 5.85	90.81 ± 8.41
Recall (%)	95.95 ± 4.97	97.75 ± 4.84	93.63 ± 7.39	87.63 ± 9.48
Specificity (%)	94.48 ± 6.56	97.48 ± 5.52	93.76 ± 5.84	87.98 ± 9.45
F1-score (%)	95.95 ± 4.97	97.55 ± 4.84	93.63 ± 7.39	95.32 ± 3.26

**Table 9:** A comparative study with recent works in case 1 of the Boon dataset (A vs. E).

Work	Main Methodology	Evaluation Metrics (%)				
		Accuracy	Precision	Recall	Specificity	F1-Score
Chen et al. [8]	DWT-ENTROPIES-CNN	99.30	100.00	98.62	100.00	/
Buldu et al. [18]	CWT-Resnet-101	99.00	99.40	99.20	/	99.30
	CWT-Resnet-50	99.60	99.50	99.70	/	99.60
	CWT-AlexNet	98.30	98.30	98.40	/	98.30
	CWT-GoogLeNet	99.60	99.50	99.70	/	99.60
	CWT-VGG-19	83.30	85.00	86.40	/	85.70
Sun et al. [19]	HG-SANet	100.00	100.00	100.00	/	/
Varlı and Yılmaz [22]	2D-CNN-CWT-LSTM	99.81	99.81	99.81	99.81	99.81
	2D-CNN-STFT-LSTM	96.50	96.36	97.01	96.41	97.01
Cao et al. [23]	CNN-Bi-LSTM	99.50	100.00	99.01	100.00	99.50
Huang et al. [25]	TCN-SA	97.37	99.91	94.88	99.91	97.30
Zang et al. [31]	LRR-TML	97.85	97.87	97.72	/	/
This work	DySC-MDE	96.50	96.36	97.01	96.41	97.01

**Table 10:** A comparative study with recent works in case 2 of the Boon dataset (C vs. E).

Work	Main Methodology	Evaluation Metrics (%)				
		Accuracy	Precision	Recall	Specificity	F1-Score
Chen et al. [8]	DWT-ENTROPIES-CNN	99.90	99.81	100.00	99.80	/
Buldu et al. [18]	CWT-Resnet-101	99.80	99.80	99.60	/	99.70
	CWT-Resnet-50	99.30	99.40	99.20	/	99.30
	CWT-AlexNet	98.30	98.30	98.40	/	98.30
	CWT-GoogLeNet	98.70	98.30	99.10	/	98.70
	CWT-VGG-19	80.00	72.00	78.30	/	75.00
Sun et al. [19]	HG-SANet	99.50	99.50	99.55	/	/
Varlı and Yılmaz [22]	2D-CNN-CWT-LSTM	99.09	99.11	99.09	99.09	99.09
	2D-CNN-STFT-LSTM	99.62	99.63	99.62	99.62	99.62
Cao et al. [23]	CNN-Bi-LSTM	99.75	99.83	99.69	99.84	99.74
Zang et al. [31]	LRR-TML	98.61	98.65	98.21	/	/
This work	DySC-MDE	97.50	97.30	97.58	97.29	97.58

**Table 11:** A comparative study with recent works in case 3 of the Boon dataset (ABCD vs. E).

Work	Main Methodology	Evaluation Metrics (%)				
		Accuracy	Precision	Recall	Specificity	F1-Score
Chen et al. [8]	DWT-ENTROPIES-CNN	98.47	96.74	95.80	99.16	/
Zaid et al. [20]	DNN(simple)-FFT	98.23	/	94.71	99.12	/
	1D-CNN(complex)-FFT	98.91	/	96.43	99.54	/
	1D-CNN(moderate)-FFT	99.13	/	98.32	99.34	/
Varlı and Yılmaz [22]	2D-CNN-CWT-LSTM	99.42	99.42	99.42	98.64	99.42
	2D-CNN-STFT-LSTM	98.20	98.20	98.20	98.99	98.09
Cao et al. [23]	CNN-Bi-LSTM	98.93	99.46	99.23	97.85	99.34
Zhu et al. [24]	STFT-TLG	99.75	99.75	98.75	98.75	99.74
This work	DySC-MDE	96.80	96.24	94.47	95.03	94.47

**Table 12:** A comparative study with recent works in case 4 of the Boon dataset (AB vs. CD vs. E).

Work	Main Methodology	Evaluation Metrics (%)				
		Accuracy	Precision	Recall	Specificity	F1-Score
Buldu et al. [18]	CWT-Resnet-101	99.30	99.30	99.40	/	99.30
	CWT-Resnet-50	98.20	98.40	98.20	/	98.30
	CWT-AlexNet	98.00	98.40	98.60	/	98.30
	CWT-GoogLeNet	96.10	95.40	96.60	/	96.00
	CWT-VGG-19	92.70	91.40	93.60	/	92.50
Sun et al. [19]	HG-SANet	98.20	98.00	98.56	/	/
Varlı and Yılmaz [22]	2D-CNN-CWT-LSTM	97.30	97.31	97.30	98.35	97.30
	2D-CNN-STFT-LSTM	98.20	98.20	98.20	98.99	98.09
Cao et al. [23]	CNN-Bi-LSTM	95.71	97.05	94.90	96.80	95.91
Zhu et al. [24]	STFT-TLG	98.75	98.75	98.33	99.17	98.74
Fu et al. [30]	GA-DCNN	93.00	90.00	90.60	93.00	90.00
This work	DySC-MDE	93.80	92.47	92.69	92.38	96.83

**Table 13:** A comparative study with recent works in the CHB-MIT dataset.

Work	Main Methodology	Evaluation Metrics (%)				
		Accuracy	Precision	Recall	Specificity	F1-Score
Cao et al. [23]	CNN-Bi-LSTM	98.43	99.14	97.84	99.21	98.39
Zhu et al. [24]	STFT-TLG	97.30	97.03	98.24	97.27	97.80
Chuang et al. [37]	Stacked 2D-CNN (18 channels)	98.47	/	100.00	98.47	/
	Stacked 2D-CNN (4 channels)	97.73	/	97.05	97.72	/
	Stacked 2D-CNN (single channel)	94.93	/	97.69	94.92	/
Shah et al. [39]	RNN-DWT	93.27	/	90.10	96.53	/
	RNN-FFT	84.60	/	79.17	89.60	/
Zhu et al. [40]	SE-CNN-BiGRU	93.70	/	91.57	98.29	83.98
	SE-TCN-BiGRU (with noise)	91.48	/	91.60	97.67	84.07
	SE-TCN-BiGRU	93.78	/	93.31	92.65	85.55
This work	DySC-MDE	98.93	98.94	98.87	98.89	98.87

## Data Availability

The datasets generated and/or analyzed during the current study are available at https://github.com/zyzc75/DySC-DME (accessed on 19 June 2025).
